# *Clostridium septicum* Aortitis and Cecal Adenocarcinoma

**DOI:** 10.1155/2010/121728

**Published:** 2010-03-07

**Authors:** Brian Moseley, Nicola W. Mwirigi, Juan Bowen

**Affiliations:** ^1^Department of Neurology, Mayo Clinic Rochester, 200 First Street SW, Rochester, MN 55905, USA; ^2^Department of Internal Medicine, Mayo Clinic Rochester, 200 First Street SW, Rochester, MN 55905, USA

## Abstract

*Clostridium septicum* aortitis is a rare infection that is highly associated with underlying malignancy. We present a case of an 82-year-old male diagnosed with both *C. septicum* aortitis and a high-grade cecal tubulovillous adenoma. The patient was offered aortic resection; however, he opted for only suppressive antibiotic therapy and a right hemicolectomy with ileocolonic anastomosis. He ultimately passed away 75 days following admission. The authors report on the connection between *C. septicum* aortitis and malignancy. The authors also discuss the need for prompt treatment with antibiotics once the infection is identified and the consideration of aortic resection given the risk of aneurysmal change with aortic dissection or rupture.

## 1. Introduction

*Clostridium septicum* is a rare infection, accounting for only 1.3% of all clostridial infections [1]. When present, *C. septicum* often colonizes locations affected by atherosclerosis (such as the aorta). This can potentially explain its predilection for the elderly; of 27 previously reported cases of *C. septicum* aortitis, all but one occurred in patients older than 55 [2]. Up to 85% of such infections are associated with an occult malignancy [3]. When infection with *C. septicum* is diagnosed, a search for an underlying neoplasm should be undertaken. Such infections also require prompt treatment with intravenous antibiotics once identified. Given the risk of aneurysmal change with aortic dissection or rupture in cases of *C. septicum* aortitis, aortic resection should be strongly considered. We present a case of *C. septicum* aortitis associated with a high-grade cecal tubulovillous adenoma. This patient consented to suppressive antibiotic therapy and a right hemicolectomy with ileocolonic anastomosis. However, he declined aortic resection. He passed away 75 days after admission.

## 2. Case Presentation

An 82-year-old man with a past medical history of hypertension, atrial fibrillation, coronary artery disease, type 2 diabetes mellitus, and chronic obstructive pulmonary disease (COPD) was admitted for treatment of a presumed COPD exacerbation. He noted fevers, chills, and fatigue for the 24 hours preceding hospitalization. On admission, he was febrile with a temperature of 39.3°C; his vital signs were otherwise stable. Physical examination revealed a distended abdomen with hypoactive bowel sounds and an irregularly irregular cardiac rhythm. Overnight he became increasingly dyspneic (respiratory rate 52) and tachycardic (heart rate 118), necessitating transfer to the Medical Intensive Care Unit (MICU).

**Figure 1:**
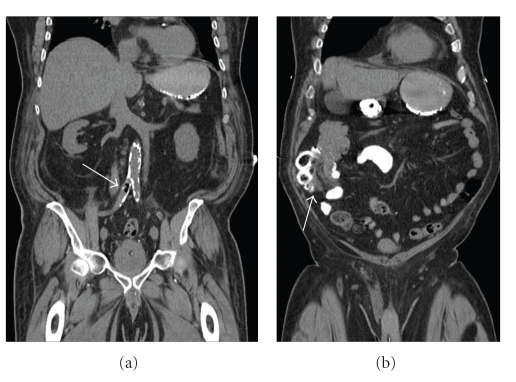
CT abdomen/pelvis (coronal): (a) multiple bubbles of gas within the lumen of the infrarenal aorta and right common iliac artery, consistent with bacterial aortitis and (b) ileo-colic intussusception caused by a cecal tubulovillous adenoma.

Within 8 hours of admission, blood cultures grew a large Gram positive bacillus, which was later identified as *Clostridium septicum*. A CT abdomen performed in the MICU revealed multiple bubbles of gas within the lumen of the infrarenal aorta and right common iliac artery (Figure 1(a)). These changes were consistent with bacterial aortitis. The study also revealed ileo-colic intussusception and a soft-tissue mass within the lumen of the ascending colon (Figure 1(b)). A high-grade cecal tubulovillous adenoma was found at colonoscopy.

Although surgical correction of his aortitis was strongly recommended, the patient declined. He opted for a right hemicolectomy with ileocolonic anastomosis (due to the development of colonic obstruction) and prolonged antibiotic therapy. The patient underwent 8 weeks of intravenous antibiotic therapy. He was initially started on Zosyn (in addition to empiric Vancomycin and Levofloxacin given the possibility of a COPD exacerbation); this was changed to Ertapenem to permit easier dosing on planned discharge to a skilled nursing facility. Given the development of an elevated alkaline phosphatase and concern about cholestasis, this was subsequently switched to IV Cefepime and Metronidazole. This was to be followed by lifelong suppressive therapy with oral amoxicillin. Serial CT imaging of the patient's abdomen while on antibiotics revealed resolution of air within the aorta and right common iliac artery. Serial imaging also revealed no evidence of mycotic aneurysm, emphysema, or dissection of the aorta. The patient's post operative course was complicated by a non-ST-elevation myocardial infarction and oliguric kidney injury requiring intermittent dialysis. He was discharged on hospital day 46. He ultimately passed away 75 days following admission. Autopsy suggested ischemic heart disease as the immediate cause of death; severe calcific coronary atherosclerosis (grade 4 stenoses of the left main coronary, left anterior descending, left circumflex, and right coronary arteries) was documented. At the time of autopsy, no evidence of residual infection was discovered.

## 3. Discussion

This case illustrates the strong association between *C. septicum* aortitis and gastrointestinal neoplasia. In a review of 162 cases of *C. septicum* aortitis, Kornbluth et al. found 81% of patients having an underlying malignancy. Colon carcinoma was discovered in 34% of patients, while a hematologic malignancy was discovered in 40% [4]. The association between this organism and gastrointestinal neoplasia may be secondary to the favorable environment afforded by these tumors. Ileocecal tumors are characterized by a low pH and a low propensity for oxidation-reduction reactions. These are two conditions which may allow *C. septicum* to flourish [5]. Mucosal ulceration within the tumor likely allows *C. septicum* to disseminate [6].

Prompt antibiotic treatment is necessary once this infection is identified. Intravenous penicillin G, third or fourth-generation cephalosporins, metronidazole, imipenem, and vancomycin have been shown to be effective against *C. septicum* [6]. Lifelong antibiotic therapy has been advocated by some, particularly in cases where a primary source of infection cannot be identified [7]. 

Aortic resection should be considered in all patients because of the risk of aneurysmal change. Aneurysms of the thoracic and abdominal aorta and iliac arteries have been associated with a rupture rate of 85% [8]. Initially, patients can present with a normal-sized aorta. However, life-threatening aneurysms can develop rapidly, sometimes within days to weeks [9]. In patients with a positive Gram stain or purulence, excision of the aneurysm with an extra-anatomic bypass is advised. For patients with a negative Gram stain and no evidence of purulence, in-situ graft reconstruction with synthetic material can be undertaken [10]. Concomitant resections of a *C. septicum* aortic aneurysm and colon cancer have also been successfully performed. Simultaneous resection is theorized to reduce hematogenous spread of *C. septicum* from unresected colonic neoplasm to the new bypass or graft [11].

Failure to treat *C. septicum* aortitis with operative management can portend a poor prognosis. In a review of 26 cases of *C. septicum* aortitis, Seder et al. found a 6-month mortality rate of 100% in those who did not undergo operative intervention. In contrast, a 6-month survival rate of 75% was documented in those patients who underwent in-situ grafting [6]. If aortic resection is not performed, serial imaging of the aorta is needed to monitor for aneurysmal transformation. Although our patient's autopsy suggested ischemic heart disease as the cause of death, one cannot confidently conclude that he would have avoided recurrence of *C. septicum* infection had he lived more than 75 days from his admission.

## References

[1] Bodey GP, Rodriguez S, Fainstein V, Elting LS (1991). Clostridial bacteremia in cancer patients: a 12-year experience. *Cancer*.

[2] Yang Z, Reilly SD (2009). *Clostridium septicum* aortitis causing aortic dissection in a 22-year-old man. *Texas Heart Institute Journal*.

[3] Alpern RJ, Dowell VR (1969). *Clostridium septicum* infections and malignancy. *Journal of the American Medical Association*.

[4] Kornbluth AA, Danzig JB, Bernstein LH (1989). *Clostridium septicum* infection and associated malignancy. Report of 2 cases and review of the literature. *Medicine*.

[5] Schaaf RE, Jacobs N, Kelvin FM (1980). *Clostridium septicum* infection associated with colonic carcinoma and hematologic abnormality. *Radiology*.

[6] Seder CW, Kramer M, Long G (2009). *Clostridium septicum* aortitis: report of two cases and review of the literature. *Journal of Vascular Surgery*.

[7] Sailors DM, Eidt JF, Gagne PJ (1996). Primary *Clostridium septicum* aortitis: a rare cause of necrotizing suprarenal aortic infection: a case report and review of the literature. *Journal of Vascular Surgery*.

[8] Müller BT, Wegener OR, Grabitz K (2001). Mycotic aneurysms of the thoracic and abdominal aorta and iliac arteries: experience with anatomic and extra-anatomic repair in 33 cases. *Journal of Vascular Surgery*.

[9] Takano H, Taniguchi K, Kuki S, Nakamura T, Miyagawa S, Masai T (2003). Mycotic aneurysm of the infrarenal abdominal aorta infected by *Clostridium septicum*: a case report of surgical management and review of the literature. *Journal of Vascular Surgery*.

[10] Messa CA, Kulkarni M, Arous EJ (1995). Double clostridial mycotic aneurysms of the aorta. *Cardiovascular Surgery*.

[11] Mohamed HK, Elliott BM, Brothers TE, Robison JG (2006). Suprarenal *Clostridium septicum* aortitis with rupture and simultaneous colon cancer. *Annals of Vascular Surgery*.

